# Specific Dynamic Parameters: A Novel Multi-View Stereo Vision Measurement System for Vector Nozzle

**DOI:** 10.3390/s26010093

**Published:** 2025-12-23

**Authors:** Zhixiao Lin, Kechen Song, Han Zhang, Zhenbo Zhou, Yansong Zhang, Chenggang Li, Yunhui Yan

**Affiliations:** 1 School of Mechanical Engineering and Automation, Northeastern University, Shenyang 110819, Chinayanyh@neu.edu.cn (Y.Y.); 2 AECC Shenyang Engine Research Institute, Shenyang 110004, China; 3 Taihang National Laboratory, Chengdu 610299, China

**Keywords:** vector nozzle, multi-view stereo vision, point cloud segmentation, fitting and measuring

## Abstract

As a crucial component of aero-engines, the accurate measurement of the motion parameters of the vector nozzle is of great significance for thrust vector control and flight performance improvement. This paper designs a dual-bearing rotating nozzle to simulate the motion of a real vector nozzle and a multi-view vision platform specifically for measuring the motion parameters of the vector nozzle. To address the parameter measurement problem, this paper proposes a technical solution of “3D reconstruction-point cloud segmentation-fitting measurement”. First, a deep learning-based multi-view stereo vision method is used to reconstruct the nozzle model in 3D. Then, the point transformer method is used to specifically extract the point cloud of the nozzle end face region. Finally, the RANSAC method is used to fit a spatial circle to the end face point cloud for measurement purposes. This paper verifies the effectiveness of the proposed method through multiple sets of comparative experiments and evaluates the reliability and stability of the measurement system using analysis of variance in measurement system analysis. The final analysis results show that the GRR% and NDC of the measurement system are 2.81% and 7.69%, respectively, and 1.84%, 50, 18, and 76, respectively, indicating that the measurement system has high accuracy and stability.

## 1. Introduction

Vectoring nozzles are an important component of aero-engines [1,2,3], and their main function is to guide and accelerate the airflow ejected from the engine to generate thrust. The magnitude and direction of thrust are controlled by changing the nozzle opening and angle. This not only improves the aircraft’s maneuverability at high angles of attack and low speeds, but also helps pilots perform many difficult and tactically valuable maneuvers, significantly increasing the aircraft’s tactical flexibility and flight stability. To ensure the control accuracy and reliability of vectoring nozzles under complex operating conditions, non-contact measurement of their geometric parameters (such as deflection angle and opening) has become a core requirement in design verification, health monitoring, and closed-loop control [4,5].

In the field of vector nozzle geometric parameter measurement, existing measurement methods mainly include: contact sensor/measurement platform-based [6], geometric modeling based on laser ranging or structured light scanning [7], and indirect inversion methods combining geometric modeling and CFD/pressure measurement [8,9]. Zhang et al. [10] used a laser tracker to perform static geometric measurement of the thrust line of a rocket engine. Although it has high accuracy, the equipment cost is high and the deployment is complicated. For vision methods, Guo et al. [11] designed a vision measurement system based on nozzle rotation axis modeling. By extracting image marker points and the spatial transformation relationship, the nozzle pitch and yaw angles are estimated. Cui et al. [12] used binocular stereo vision and the geometric constraint model to improve measurement stability, providing a new path for non-contact measurement.

Despite the progress made in visual measurement accuracy, some problems remain. Contact measurement devices can affect the movement of vector nozzles and reduce measurement accuracy under vibration conditions. Laser ranging devices are complex to deploy and susceptible to occlusion. While camera calibration methods have the advantage of being non-contact, they are not suitable for vector nozzle scenarios with sparse surface textures and complex structures. They also lack a complete 3D reconstruction process, resulting in incomplete structural perception. Furthermore, their robustness to high-frequency deformation and multi-part coordinated motion scenarios needs improvement [13,14,15]. Therefore, we must carefully consider the following issues.

1. How do we use non-contact measurement methods and adapt to the dynamic changes of the nozzle at the same time? This includes the joint calibration of multiple cameras and the perception of the nozzle structure.
2. How do we specifically extract the geometric parameters of the nozzle? These include the nozzle opening size, nozzle deflection angle, and azimuth angle.

In response to the above problems and research gaps, this paper proposes a “3D reconstruction-point cloud segmentation-fitting measurement” technical solution. A dual-bearing rotating nozzle is designed to simulate the motion of a real vector nozzle, and a multi-view imaging platform is built to capture multi-view images as data sources. First, the intrinsic and extrinsic parameters of the multi-view camera are estimated using the Structure-from-Motion method, and a deep learning-based multi-view stereo vision method is employed to achieve high-precision dense reconstruction of the nozzle structure, obtaining complete point cloud data. Subsequently, a Transformer-based point cloud segmentation network is used to accurately segment the end face points. Finally, the RANSAC circle fitting algorithm and spatial geometry theory are used to accurately measure the nozzle opening and deflection angle. This paper proposes a specific dynamic parameter extraction method based on multi-view vision. This method can fully perceive the nozzle structure during 3D reconstruction and ultimately specifically extract motion parameters such as nozzle opening and deflection angle. The proposed method is not limited to the measurement of motion parameters of vector nozzles, but can also be extended to other complex and movable components, such as adjustable air intakes and deflection surfaces, providing a high-precision and scalable solution for the measurement of motion parameters of other complex structures.

In summary, the main contributions of this paper are summarized as follows.

1. A technical solution of “3D reconstruction–point cloud segmentation–fitting measurement” was proposed in this paper, which innovatively realizes “non-contact, no additional, and calibration-free” visual measurement of the motion geometry parameters of vector nozzles.
2. An axisymmetric vector nozzle model was designed and a multi-view vision platform was built in this paper, through which the dynamic geometric parameters of the vector nozzle can be obtained by three methods: drive motor parameter calculation, laser sensor, and vision measurement, thereby achieving the measurement of the accuracy and consistency of the vision measurement method.
3. AprilTag-encoded landmarks were used in this paper to address the lack of absolute scale information in the vector nozzle point cloud obtained using multi-camera stereo vision. The complex multi-camera joint calibration process was replaced, reducing the complexity of the visual measurement method and the requirements for the test environment.

## 2. Related Works

### 2.1. Thrust Vector Nozzle

Vector nozzle technology is an essential technology for aero-engines, which has greatly improved the performance of aircraft, specifically by improving the aircraft’s maneuverability and agility, reducing the aerodynamic control surfaces of the aircraft, reducing the tail fin, etc. [16,17,18]. Since the 1970s, various countries around the world have conducted extensive research on thrust vector nozzle technology. In 1973, Pratt Whitney of the United States proposed an axisymmetric convergent nozzle [19]. The nozzle can swing up and down in the pitch plane relative to the axis of rotation, thereby obtaining additional aircraft control torque. The advantages of this device are simple structure, good airtightness, and simple and reliable vector control. In 1986, Pratt Whitney of the United States designed a thrust vector nozzle with pitch, yaw and reverse thrust, namely the spherical convergent adjustment vane nozzle [20]. The pitch vector angle of the nozzle reaches ±30°, the yaw vector angle reaches ±20°, and the steering is flexible. The American GE company initially researched axisymmetric thrust vectoring nozzles that can rotate 360°—the axisymmetric vectoring nozzle (AVEN) [21]. By deflecting the expansion section of the nozzle in all directions, the thrust of the nozzle is vectored in all directions. It retains the good aerodynamic performance of the previous axisymmetric expansion nozzles, and expands the function of the expansion section in the connection, so that it can generate ultrasonic airflow and deflect the airflow direction according to the needs of the aircraft [22].

Following research, this paper designed a dual-bearing rotating nozzle model capable of simulating the angle changes and nozzle opening and closing of a vector nozzle. The nozzle model can achieve omnidirectional deflection with a vector deflection angle $\alpha \leq 45^{{}^{\circ}}$. and a vector azimuth angle $0^{\circ }\leq \beta \leq 360^{\circ }$, with a maximum nozzle end face diameter of 270 mm. A laser rangefinder and angle calculation are used to accurately obtain the actual nozzle deflection angle and opening size.

**Figure 1:**
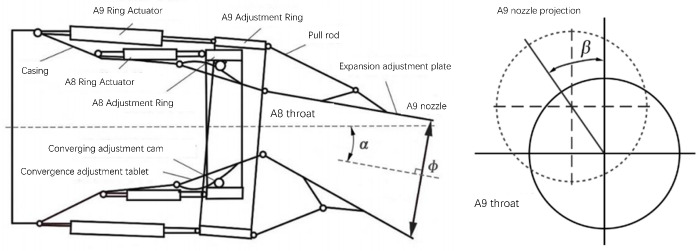
Definition diagram of vector nozzle motion parameters.

The definition of the motion parameters of the vector nozzle is shown in Figure 1. The attitude of the A9 nozzle end face in the vectoring nozzle directly controls the magnitude and direction of the aero-engine thrust vector. This attitude can be fully described by three motion parameters: nozzle diameter φ, vector deflection angle α, and vector azimuth angle β. The nozzle diameter φ is the diameter of the approximately circular A9 nozzle end face formed by the ends of multiple expanding vanes. The nozzle diameter φ directly determines the nozzle exit area, thus affecting the matching relationship between airflow velocity and mass flow rate, and controlling the engine thrust. The vector deflection angle α is the angle between the normal to the A9 nozzle end face and the normal to the A8 throat surface. The vector deflection angle α reflects the magnitude of the thrust vector deviating from the engine axis, i.e., the magnitude of the lateral force. As α increases, the engine can generate greater lateral force and torque, assisting the aircraft in maneuvering. The thrust vector azimuth angle β is the angle between the projection of the normal to the A9 nozzle end face onto the A8 throat surface and the positive pitch direction of the aircraft. It varies within the range of −180° to 180°, and its sign is determined using the right-hand rule based on the normal to the A8 throat surface. The thrust vector azimuth angle β reflects the direction in which the thrust vector deviates from the engine axis, i.e., the direction of the lateral force. Assuming $\alpha \neq 0^{\circ }$, when $\beta \neq 0^{\circ }$, the lateral force is upward along the pitch direction; when $\beta =90^{\circ }$, the lateral force is to the left along the horizontal direction.

### 2.2. Multi-View Stereo Vision Measurement

Multi-view stereo vision measurement technology acquires images of the object to be measured from different angles using a visual sensor [23], obtains target information from different angles, and then performs stereo matching based on a 3D model to complete the measurement task. Among them, multi-view stereo vision measurement methods are based on monocular and binocular vision and can be composed of multiple monocular or binocular systems. The basic principle of binocular stereo vision is similar to the imaging system of the human eye [24], which consists of two cameras located on the same plane, with parallel optical axes and the same height. With the development of deep learning, deep learning-based multi-view stereo vision methods have become mainstream. In 2018, Yao et al. proposed an end-to-end deep learning architecture called MVSNet [25], whose core lies in learning feature extraction, cost volume construction and depth regularization strategy. Subsequently, researchers have made many improvements to these core processes [26,27,28]. In recent years, the Transformer structure has been introduced into the field of multi-view stereo vision, such as MVSFormer [29], which achieves more effective cross-view information fusion by constructing a feature pyramid and a view attention mechanism. MVSFormer++ [30] combines a pre-trained visual Transformer (ViT) and a feature pyramid network (FPN) to demonstrate superior performance in multi-scale feature extraction and cost volume aggregation, significantly improving the structural perception and robustness of reconstruction, and is particularly suitable for reconstruction scenarios with complex backgrounds and sparse textures. However, current mainstream multi-view stereo vision methods focus on improving the accuracy of disparity estimation, rather than on the scale consistency and measurability of reconstruction results, which has limitations when facing application scenarios such as engineering measurement. To this end, this paper uses AprilTag encoding marker points [31] on the basis of high-precision dense reconstruction of multi-view stereo vision, which solves the problem that the vector point cloud obtained by multi-view stereo vision methods does not have absolute scale information. This provides a reliable foundation for subsequent point cloud segmentation and fitting measurement.

### 2.3. Point Cloud Segmentation

Three-dimensional point clouds are an important means of understanding spatial structure and are widely used in fields such as autonomous driving, embodied intelligence and industrial geometric measurement. According to different processing methods, existing segmentation methods are mainly divided into the following three categories: point-level methods, voxelization methods, and projection and attention methods. PointNet [32] first proposed to directly process point cloud coordinates with the multilayer perceptron, which has permutation invariance. Then, PointNet++ [33] introduced hierarchical sampling and neighborhood aggregation, which improved the local structure modeling ability. Voxelization methods convert point clouds into regular grids, which facilitates the extraction of features using standard convolution operations. For example, VoxelNet [34] converts point clouds into regular voxel grids and extracts spatial features through three-dimensional convolution. SparseConvNet [35] and MinkowskiNet [36] and other methods use sparse convolution operations to effectively reduce redundant computation. Zhao et al. [37] proposed a multi-task network with PointNet as a shared encoder and three parallel branches, and proved that the shared encoder and joint training can improve the segmentation robustness and overall preprocessing efficiency in noisy scenes. In recent years, the Transformer structure has been introduced into the field of point cloud semantic understanding. The Point Transformer series [38] has introduced an attention mechanism to significantly improve the ability to model semantic dependencies between points. GPSFormer [39] proposed the Global Perception Module (GPM) and Local Structure Fitting Convolution (LSFConv), which take into account both local geometric details and global semantic information. Based on Point Transformer, this paper uses the nozzle point cloud face as a label to create a dataset. Based on the S3DIS pre-trained weights, it fine-tunes the weights and combines them with the Focal Loss training strategy to segment the vector nozzle end face point cloud.

### 2.4. Fitting Measurement

Geometric fitting of three-dimensional point clouds is a key step in extracting structural parameters and achieving precise measurement. Taubin circle fitting [40] and Pratt fitting method [41] are widely used in circle/ellipse parameter estimation, but they are all circle fitting methods applied to two-dimensional planes and are not directly applicable to the fitting of circular structures in three-dimensional point clouds. RANSAC [42] is the most widely used robust fitting method, which is suitable for vector nozzle end face measurement tasks. After completing point cloud segmentation, this paper uses the RANSAC fitting algorithm to perform circle fitting on the nozzle end face and background plate, and then extracts geometric parameters such as nozzle opening and deflection angle to achieve a high-precision and robust automated measurement process.

## 3. Proposed Method

**Figure 2:**
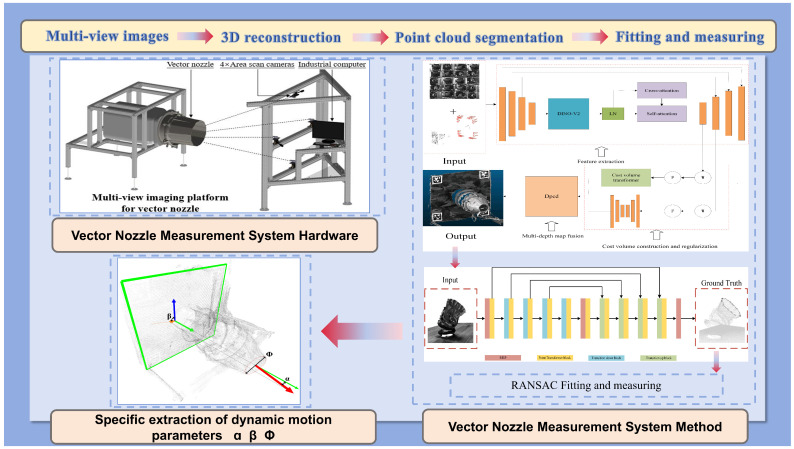
Overall framework diagram.

This section primarily introduces our method for measuring the motion parameters of a vector nozzle. As shown in Figure 2, firstly, this paper adopts a three-dimensional nozzle reconstruction method based on multi-view stereo vision, while introducing AprilTag visual markers as a point cloud scale reference. Subsequently, this paper uses a point Transformer point cloud segmentation network to automatically extract the nozzle end face region, and finally, based on the RANSAC fitting algorithm, achieves high-precision measurement of the vector nozzle nozzle opening, deflection angle, and azimuth angle.

### 3.1. 3D Reconstruction

**Figure 3:**
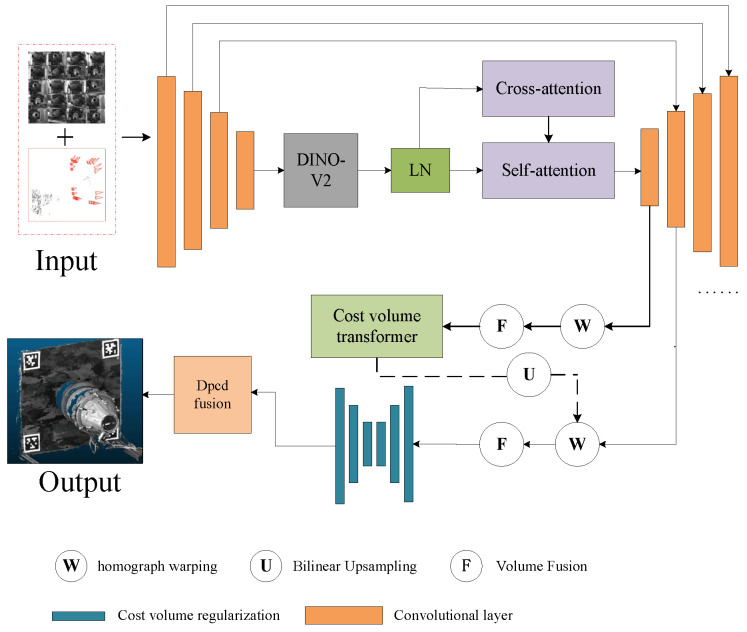
Multi-view stereo vision reconstruction network diagram.

During the motion of the vector nozzle, it is essential to fully perceive the nozzle structure and the camera pose of the scene. Therefore, this paper adopts a multi-view stereo vision-based method for 3D reconstruction of the nozzle model. First, sparse reconstruction of the scene is performed using structure from motion to obtain the poses of 20 area array cameras. Based on the camera pose information, a deep learning-based multi-view stereo vision method is then used. This paper employs a transformer-based multi-view stereo vision method, as shown in Figure 3, to obtain a 3D representation of the dense point cloud of the scene.

#### 3.1.1. Structure-from-Motion

**Figure 4:**
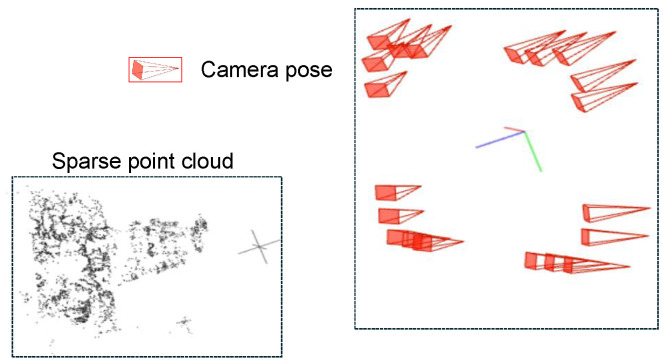
Schematic diagram of SFM results.

Structure-from-Motion starts with two initial views, estimates the relative camera pose through feature matching and 2D-2D geometric constraints, and then reconstructs spatial points using triangulation. New images are introduced using a 3D-2D PnP method, recursively performing point cloud expansion and camera pose solving. This paper uses an incremental Structure-from-Motion method implemented with COLMAP to calibrate 20 industrial cameras. To improve accuracy, COLMAP introduces Bundle Adjustment optimization to globally and jointly adjust the positions of all cameras and points. The final output includes the camera intrinsic and extrinsic parameters Ki, Ri, Ti, and a sparse 3D point cloud for each image. The results are shown in Figure 4. In the input, the left side represents the sparse point cloud of the nozzle scene, and the right side represents the poses of the 20 industrial cameras.

#### 3.1.2. Multi-View Stereo Vision

The core of the multi-view stereo vision algorithm is to first select a reference viewpoint as the baseline viewpoint based on the multi-view camera matching score obtained from Structure-from-Motion. Then, a deep learning network is used to extract features from the input image. The homography matrix Hi is used to reproject the features of the source feature image under different depth assumptions to the reference viewpoint, thus constructing the feature volume Vi from the source image to the reference image.

(1) $$x^{\prime }∼H_{i}\left(d\right)\cdot x$$

(2) $$H_{i}\left(d\right)=K_{i}\cdot R_{i}\left(I-\frac{\left(t_{l}-t_{i}\right)\cdot n_{l}^{T}}{d}\right)\cdot R_{i}^{T}\cdot K_{l}^{-1}$$

(3) $$C=\frac{\sum_{i=1}^{N}{\left(V_{i}-\bar{V}\right)}^{2}}{N}$$

The cost volume C(d) is generated by averaging and averaging the feature volumes of the N source views. 3DCNN is then used to regularize the cost volume, resulting in a three-dimensional probability volume P(d) representing the probability of a pixel being at depth d. To obtain the depth estimate of the reference view from the probability volume P, the expectation along the depth dimension is calculated. The depth estimation formula is as follows:

(4) $$d_{E}=\sum_{d=d_{\min}}^{d_{\max}}d\times P\left(d\right)$$

The Transformer-based multi-view stereo vision framework MVSFormer++ was adopted, as shown in Figure 3. This paper provides 20 input images of size 1280 × 960, along with camera parameters provided by SFM. First, multi-scale features of the input images are extracted using FPN and DINOv2. At each scale level, based on the camera parameters, homography transformation is used to align the images to the reference view according to the assumed depth, forming an aligned feature body on a multi-scale, discrete depth assumption. Based on this, a four-dimensional vector cost volume $V_{\;s}$ is constructed at this scale level, with dimensions of $H_{\;s}\times W_{\;s}\times N_{\;s}\times C_{\;s}$, where $H_{\;s}$ and $W_{\;s}$ represent the height and width of the feature map at the s-th scale level; $N_{\;s}$ represents the number of layers in the discrete depth hypothesis at the s-th scale level; and $C_{\;s}$ represents the number of feature channels at the s-th scale level. Then, at the coarsest scale, the cost volume is regularized using Cost volume transformer. The four-dimensional vector cost volume $V_{\;s}$ is regularized to obtain a three-dimensional probability volume $P_{\;s}$. Its dimension is $H_{\;s}\times W_{\;s}\times N_{\;s}$. Finally, the expectation of the three-dimensional probability volume $P_{\;s}$ is calculated along the depth dimension to obtain a depth map D (1280 × 960), and the scene dense point cloud is obtained by fusing the depth maps.

#### 3.1.3. AprilTag Scale Return

**Figure 5:**
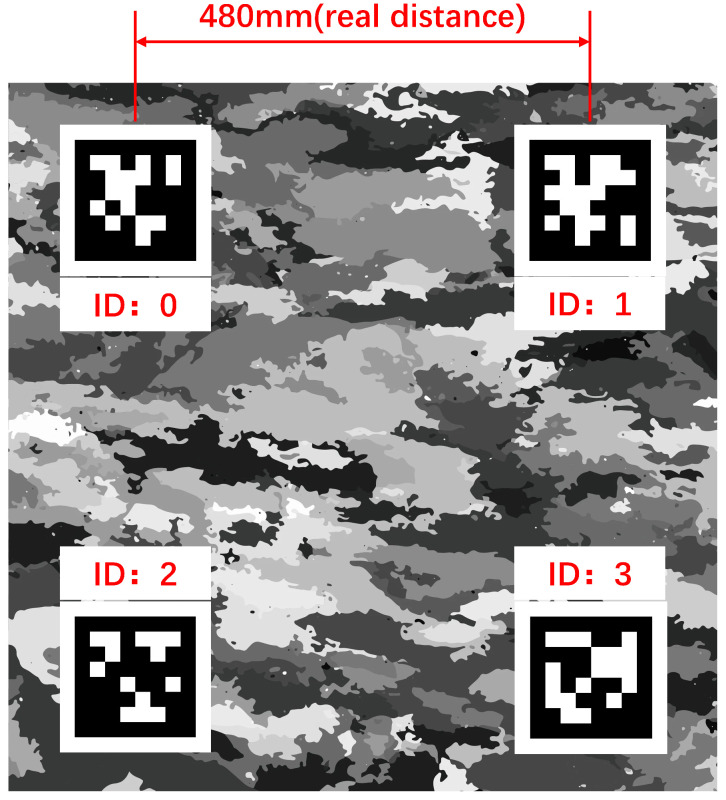
AprilTag-coded marker diagram.

Since the reconstruction results of Structure-from-Motion and multi-view vision matching only retain relative structural information and cannot provide the true physical scale, an external scale reference needs to be introduced for recovery. In this paper, four AprilTag markers with known spacing are placed on the nozzle reference plate as shown in Figure 5, which serve as the scale reference source. At the same time, this paper takes the direction of the line connecting the centers of the markers as the zero value direction of the azimuth angle β.

Considering the problems of occlusion and insufficient resolution in directly detecting marker positions in dense point clouds, this paper adopts an indirect strategy of image-space detection to 3D mapping to obtain the 3D position of markers in the point cloud, and then obtains the distance in the point cloud coordinate system. First, 2D detection is performed to detect AprilTag markers in multi-view 2D images. In the reconstruction pipeline of MVSFormer++, the 3D coordinates of the neighborhood points near the center points of the four AprilTags are determined by depth map fusion. Finally, the scale factor is obtained by calculating the physical distance between any two AprilTag centers and the Euclidean distance in the reconstructed AprilTag point cloud.

(5) $$s=\frac{D_{\mathrm{real}}}{D_{\mathrm{rec}}}$$

where Dreal is the physical distance to the center of the AprilTag; Drec is the Euclidean distance in the reconstructed point cloud of the AprilTag.

### 3.2. End Face Point Cloud Segmentation

To achieve a high degree of automation in the measurement process, manually screening end face points frame by frame in the scenario of vector nozzle motion is almost impractical. This paper adopts a transformer-based point cloud segmentation network, which can quickly and accurately identify the end face region in the point cloud, thereby realizing automated measurement.

#### 3.2.1. End Face Segmentation Dataset

To improve the generalization ability of point cloud segmentation for nozzle scenes, this paper preprocesses the dense point cloud obtained by multi-view visual matching and reconstruction and creates a point cloud segmentation dataset.

The overall preprocessing workflow is based on Open3D, and the specific steps are shown in Algorithm 1: First, it is determined whether the point cloud contains color information; if the original point cloud is in RGB format, it is converted to grayscale format to unify the data channel structure and simplify the subsequent processing. Based on the scale factor calculated in the previous section, the point cloud coordinates are uniformly scaled to make the point cloud coordinate space consistent with the actual physical space, ensuring the true scale of subsequent geometric parameter estimation. Considering that multi-view stereo matching reconstruction methods are often accompanied by redundant structures and sparse point clusters, this paper uses a density statistical method to extract the main structural regions. By statistically analyzing the neighborhood density of each point, the core region with the highest point density is cropped and retained, and the background and invalid structures are initially removed. To reduce the computational burden and maintain structural uniformity, a voxel downsampling method is then used to divide the point cloud into a fixed-size voxel grid. Points within each voxel are represented by their centroids, thereby reducing the number of points while retaining spatial distribution characteristics. Finally, statistical filtering is performed to remove outliers.

The nozzle end-face segmentation dataset is designed as follows: The data type includes three different vector nozzle models, covering various opening angles and deflection angles. This paper defines the segmentation task as a binary segmentation problem, involving nozzle end-face point clouds and other background point clouds. Both the nozzle end-face and background point clouds are semantically labeled using CloudCompare. Each point cloud contains xyz coordinates and a binary label, and after the proposed 3D reconstruction process and Open3D-based point cloud post-processing, it contains an average of approximately 100,000 valid points. The dataset contains 80 point cloud scenes, with 70% used for training, 10% for testing, and 20% for validation. This dataset is specifically designed for nozzle end-face segmentation tasks and exhibits good label quality and structural generalization ability.
**Algorithm 1** Point cloud post-processing algorithm**Require:** point Cloud, scale Factor, radius *r*, neighbors *k*, voxel size *v*, threshold**Ensure:** preprocessed Point Cloud  1:**if** point Cloud has RGB color **then**  2:    Convert point Cloud to grayscale  3:**end if**  4:**for** each point P=(x,y,z) in point Cloud **do**  5:    p←(x·scaleFactor,y·scaleFactor,z·scaleFactor)  6:**end for**  7:**for** each point *P* in point Cloud **do**  8:    Density(P)← number of neighbors of *P* within radius *r*  9:**end for** 10:Points ←{p∣Density(p)≥k} 11:**for** each point *p* in dense Points **do** 12:    voxelIndex←⌊p/v⌋ 13:    Add *p* to voxelMap[voxelIndex] 14:**end for** 15:Down sampled Points ←∅ 16:**for** each voxel Index in voxel Map **do** 17:    centroid←average(voxelMap[voxelIndex]) 18:    Add centroid to Down sampled Points 19:**end for** 20:Preprocessed Point Cloud ←∅ 21:**for** each point *p* in Down sampled Points **do** 22:    Mean Dist. ← mean distance to *k* nearest neighbors 23:    **if** Mean Dist. ≤ Threshold **then** 24:        Add *p* to Preprocessed Point Cloud 25:    **end if** 26:**end for** 27:**return** Preprocessed Point Cloud

#### 3.2.2. Point Cloud Segmentation Algorithm

**Figure 6:**
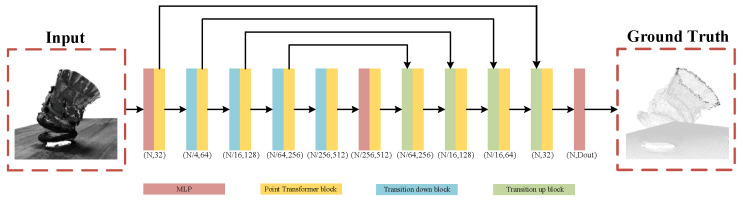
End Face Segmentation Network Diagram.

This paper adopts the Point Transformer point cloud segmentation network framework, as shown in Figure 6. Specifically, the network input is an N*C tensor, where N represents the number of points in the point cloud, and C represents the feature channels. Initially, it is a six-dimensional vector containing positional (x, y, z) information and color (r, g, b) information. In the encoder stage, as the number of layers increases, the number of points N is gradually downsampled, but the number of feature channels Ci increases accordingly. In the decoder stage, through upsampling and skip connections, the number of points N is gradually restored, and the number of feature channels gradually decreases. The final output is N*K, where K is the number of segmentation categories. In this paper, the number of categories is 2, i.e., K is 2. The network as a whole adopts an Encoder–Decoder architecture, which is particularly suitable for processing nozzle structures with obvious local geometric shapes and class imbalance in point clouds. Its core attention calculation can be formally represented as

(6) $$y_{i}=\sum_{x_{j}\in x\left(i\right)}\rho \left(\gamma (\varphi \left(x_{i}\right)-\psi \left(x_{j}\right)+\delta )\right)⊙\left(\alpha \left(x_{j}\right)+\delta \right)$$

where $x_{i}$ represents the center point, and $x_{j}$ represents the neighboring points around the center point. By simultaneously modeling local neighborhood point cloud features and global dependencies through a self-attention mechanism, the spatial structure information of the point cloud is effectively captured, thereby improving the robustness and accuracy of segmentation.

Since the nozzle end face points account for a relatively small proportion of the overall point cloud, significant class imbalance occurs during training. Therefore, this work employs a Focal Loss strategy:

(7) $$FL\left(p_{i}\right)=-\alpha \cdot {\left(1-p_{i}\right)}^{\gamma }\cdot \log\left(p_{i}\right)$$

By adjusting the class balance factor α and the focusing silver γ, the weights of the positive and negative class point clouds can be effectively balanced, and the training can be focused on the difficult-to-classify samples. The final training backend segmentation accuracy was 0.8738, and the inversion of performance (IoU) was 0.6461.

### 3.3. Fitting Measurement

#### 3.3.1. Geometric Fitting

Since the point cloud of the nozzle end face we obtained is not completely coplanar, this paper uses a two-stage RANSAC algorithm to perform spatial circle fitting on the nozzle end face.

In the first stage, RANSAC is applied to the nozzle end-face point cloud $P=\{p_{i}\}$ to obtain the best-fit plane $\pi_{c}$. Then, the nozzle end-face point cloud P is orthogonally projected onto the fitting plane $\pi_{c}$ to form a planar point set $P^{\prime }$, which is used for two-dimensional circle fitting in the second stage.

In the second stage, RANSAC is performed on the planar point set $P^{\prime }$ on the fitting plane $\pi_{c}$ to fit a two-dimensional circle. This yields the coordinates c of the center of the planar circle, the radius r, and the normal vector $n_{c}$.

Meanwhile, this paper uses a single-stage RANSAC plane fitting method to obtain the plane $\pi_{b}$ and its normal vector $n_{b}$ from the point cloud $B=\{b_{J}\}$ of the reference plate region, which serves as a reference for measuring the nozzle deflection angle and azimuth angle.

#### 3.3.2. Motion Parameter Measurement

Based on the fitting results from the previous section, the key geometric parameters of the nozzle structure can now be calculated. The nozzle opening ϕ is the diameter of the spatial circle, calculated from the radius r of the fitted circle:

(8) ϕ=2r

The deflection angle α is the angle between the nozzle normal vector $n_{c}$ and the reference plate normal vector $n_{b}$. It is calculated using the vector angle formula:

(9) $$\alpha =\arccos(\frac{n_{c}\cdot n_{b}}{∥n_{c}∥\cdot ∥n_{b}∥})$$

By calculation, key parameters such as nozzle opening and deflection angle can be reliably extracted. This module serves as the key output of the entire vision measurement system, directly measuring the geometric parameters of the vector nozzle mechanism.

## 4. Experiment and Analysis

This section mainly introduces our measurement system, experimental methods, evaluation indicators, and measurement result analysis. To verify the effectiveness of the proposed method, this paper conducted three sets of comparative experiments to measure the motion parameters of the vector nozzle under different attitudes. Finally, we used MSA to perform quantitative analysis of the measurement results based on the established evaluation indicators, and ensured the repeatability and stability of the measurement system.

### 4.1. Measurement System

**Figure 7:**
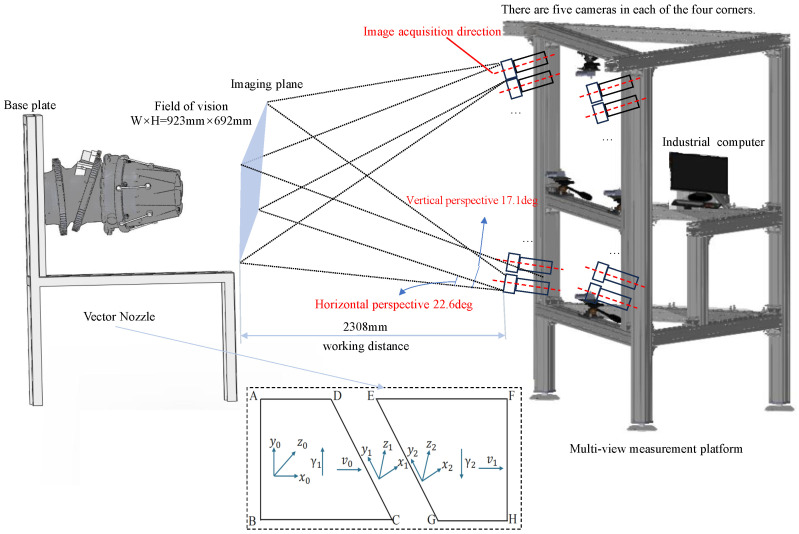
Measurement system structure diagram.

This paper constructs a dual-bearing rotating vector nozzle for simulating the accuracy test of vector nozzle motion, and designs a multi-view vision platform specifically for capturing multi-view images. The overall structure is shown in Figure 7.

**Figure 8:**
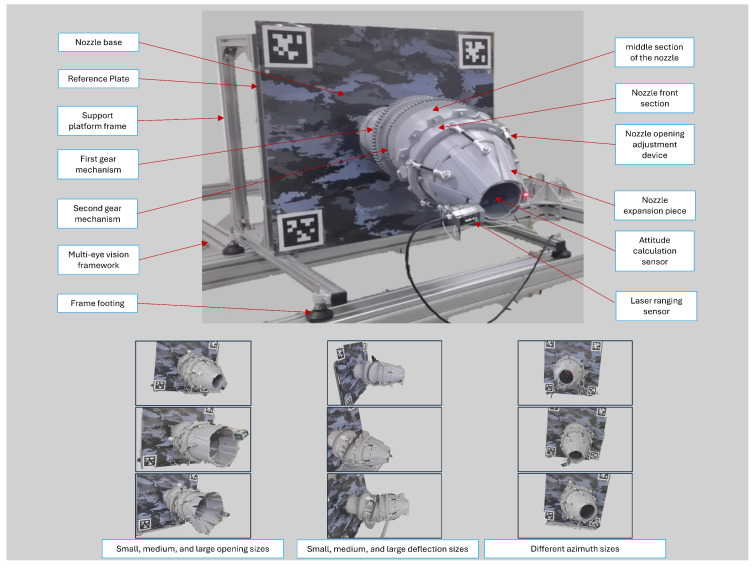
Schematic diagram of vector nozzle mechanism.

The structure of the vector nozzle is shown in Figure 8. The nozzle opening is adjusted by adjustment device, and the nozzle deflection is achieved by two sets of gear mechanisms. Both sets of gears are driven by DC motors. The motor, combined with a speed controller and forward/reverse and travel limit switches, can realize the forward/reverse rotation of the nozzle, speed regulation, and limit control. The nozzle deflection direction reset and zeroing are accomplished through two sets of photoelectric switch sensors and attitude calculation sensors.

The nozzle opening was measured using a laser rangefinder to determine the actual nozzle diameter. To obtain the true nozzle deflection angle, this paper studied the motion of the nozzle model. Based on Figure 8, we simplified the model as shown in Figure 6, where segment BD rotates around the x-axis of the absolute coordinate system, and segment EF rotates around the $x_{1}$ axis. Through coordinate transformation and geometric characteristics, we know that the angle between $v_{0}$ and $v_{1}$ represents the nozzle deflection attitude. This paper calculates the true nozzle deflection angle using the rotation angles of two gear mechanisms. The calculation method is as follows:

(10) $$\alpha =\arccos(\sin^{2}\gamma_{1}+\cos^{2}\gamma_{1}\cos\gamma_{2})$$

where $\gamma_{1}$ is the rotation angle of the rear gear, and $\gamma_{2}$ is the rotation angle of the front gear.

Meanwhile, this paper placed a reference plate directly behind the nozzle model, with its plane parallel to the plane of the rear gear bearing. The entire support platform is constructed of 2020 aluminum profiles and can be used to fix the overall nozzle model and support the motor controller and power supply. Ultimately, our designed vector nozzle model can achieve omnidirectional deflection with $\alpha \leq 45^{\circ }$ and $0^{\circ }\leq \beta \leq 360^{\circ }$.

**Figure 9:**
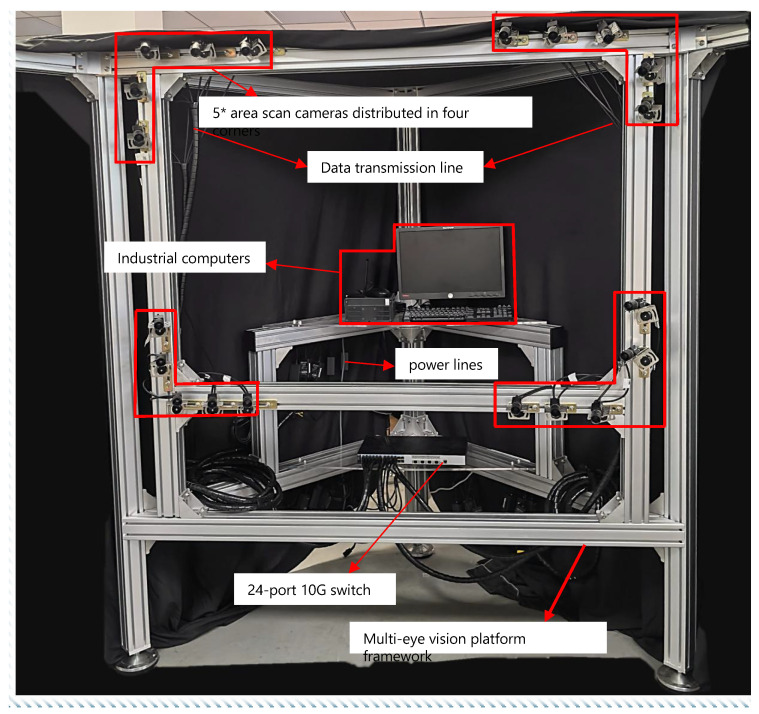
Multi-view vision platform structure diagram.

As shown in Figure 9, to achieve real-time synchronous acquisition of multi-view images of the vector nozzle, this paper built a multi-view vision platform. Considering both safety and stability, 2 mm thick 8080 European standard aluminum profiles were used as the raw material for the frame construction. The platform is equipped with 20 MV-CE013-50GM (Hangzhou Hikrobot Co., Ltd., Hangzhou, China) industrial area array cameras with MVL-MF1224M-5MPE lenses (Hangzhou Hikrobot Co., Ltd., Hangzhou, China). The cameras are stably connected to the platform via adapters, ball heads, corner brackets, and T-bolts. Simultaneously, the cameras are connected to an HIK industrial control computer via a 24-port 10 Gigabit switch (TP-Link Technologies Co., Ltd., Shenzhen, China). This paper built the imaging workflow based on Vision Master, ultimately achieving synchronous multi-view imaging.

**Figure 10:**
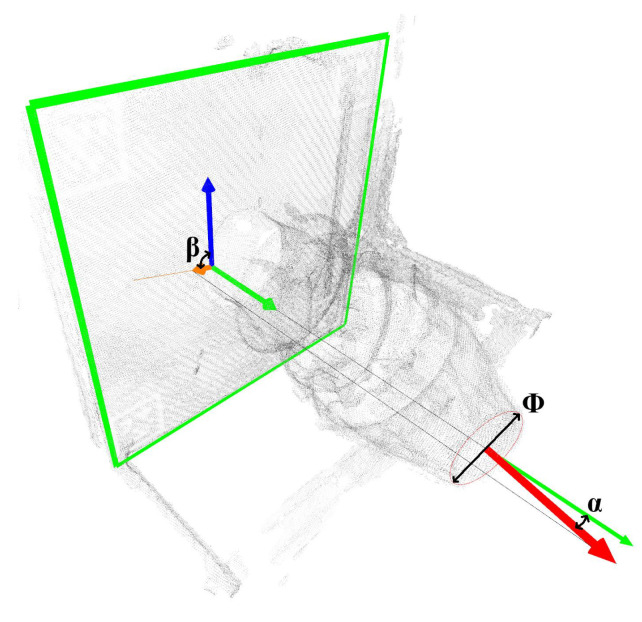
Visualization of measurement results diagram.

The experimental details are as follows: This experiment was conducted on a multi-view vision platform based on Vision Master, continuously acquiring multi-view images of the nozzle from 20 cameras simultaneously. First, the acquired multi-view images were input into a reconstruction algorithm framework to obtain a dense 3D point cloud model. Then, the reconstructed point cloud model was tested on a pre-trained point transformer network to obtain the nozzle end-face point cloud. Finally, the RANSAC method was used to calculate and obtain the nozzle’s motion parameters. All experimental algorithms in this paper were implemented on a PyTorch 2.0 framework within an Ubuntu 20.04 Python 3.8 environment. The measurement results were visualized as shown in Figure 10. Ultimately, the measurement requirements for vector nozzles were achieved using the proposed measurement method framework. In this diagram, the red circle represents the measured end face diameter, the orange arrow indicates the initial reference direction of the azimuth angle, and the blue arrow indicates the calculated azimuth angle direction; the angle between the two is the azimuth angle magnitude. The green arrow indicates the normal direction of the reference plate, and the red arrow indicates the normal direction of the vector nozzle end face circle; the angle between the two corresponds to the nozzle deflection angle.

### 4.2. Comparative Experiment

**Figure 11:**
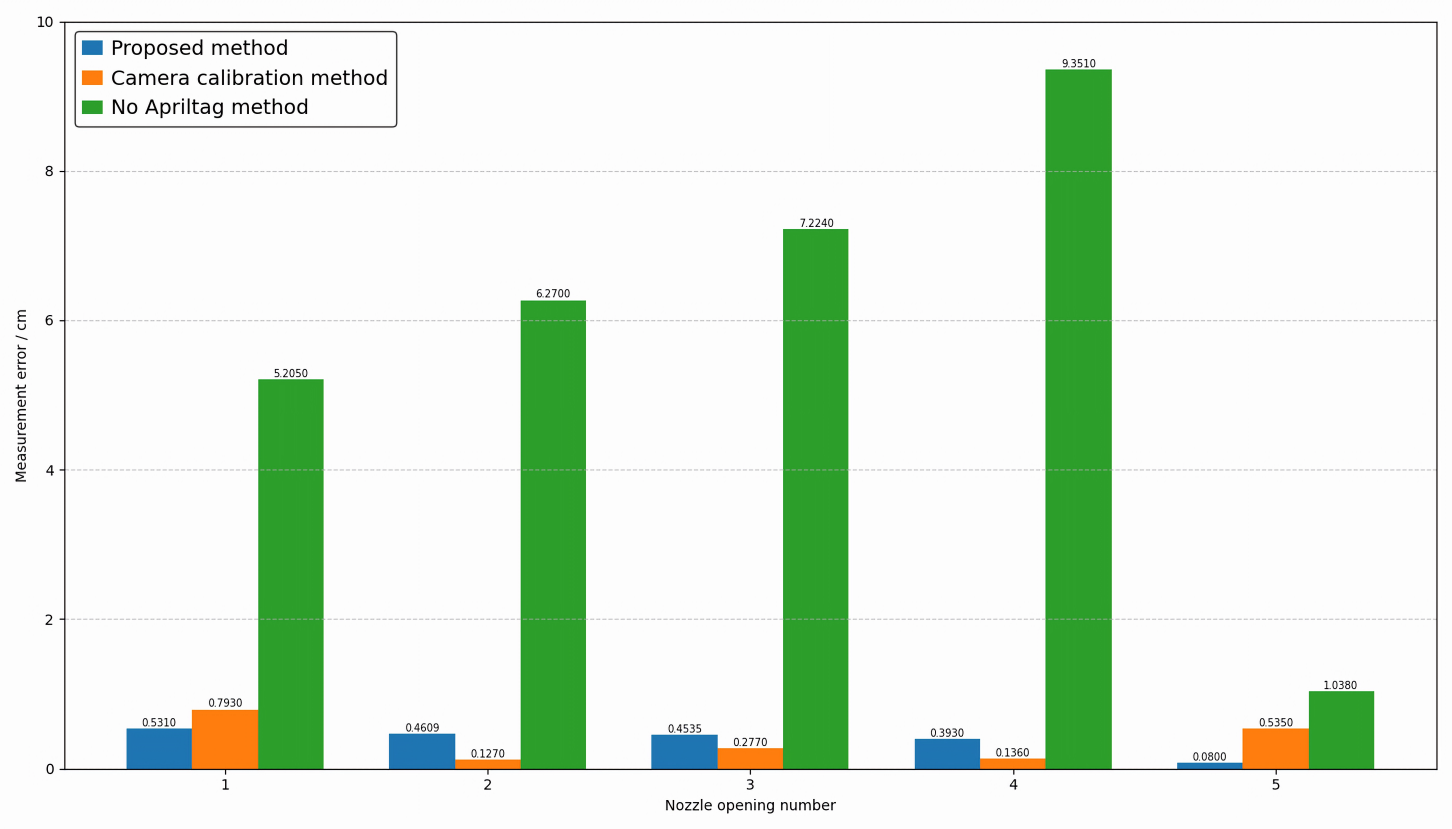
Vector Nozzle Opening Error Analysis Diagram.

**Figure 12:**
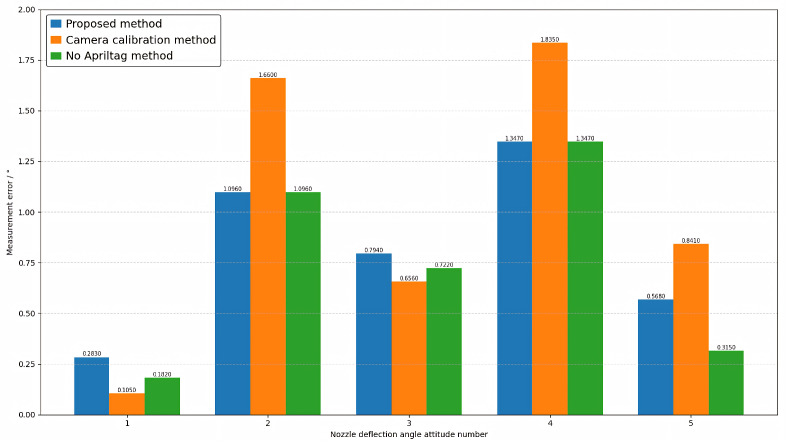
Vector Nozzle Deflection Angle Error Diagram.

**Figure 13:**
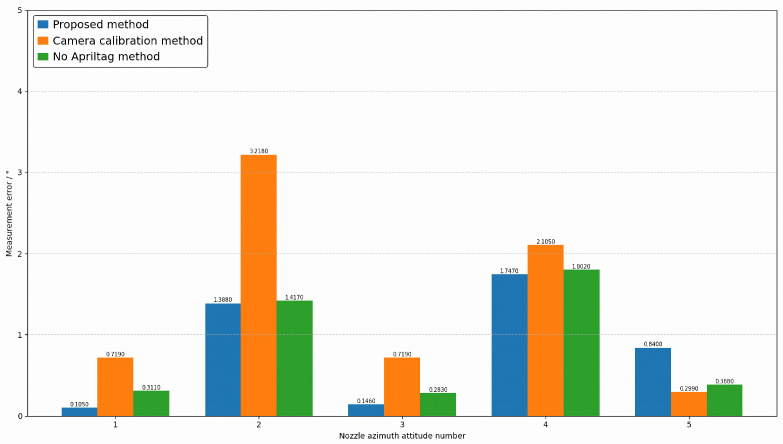
Vector Nozzle Azimuth Error Diagram.

To verify the effectiveness of the proposed method, three sets of comparative experiments were designed. The first set of experiments involved direct measurement based on our proposed method framework using multi-view vision to detect marker points. The second set involved joint calibration of 20 industrial cameras, testing the proposed method under conditions based on real camera poses. The third set measures without adding markers within the framework of the method proposed in this paper. This paper conducts experiments on the attitudes of five different nozzles, including opening angle, deflection angle, and azimuth angle. The results are shown in Figure 11, Figure 12 and Figure 13, where the horizontal axis represents the number of poses, and the vertical axis represents the measurement error. Blue represents the proposed method, orange represents the camera calibration method, and green represents the method without AprilTag marker points. Experimental results show that the proposed detection method can replace the camera calibration process while maintaining measurement accuracy, and the proposed measurement method achieves measurement accuracy with relatively small errors. This enables the measurement of vector nozzle motion parameters.

### 4.3. Evaluation Indicators and Measurement Systems Analysis

This paper uses measurement system analysis (MSA) to evaluate the experimental results. MSA can systematically analyze the performance of measurement systems, which mainly include five key characteristics: accuracy, precision, stability, linearity, and resolution. Different analysis methods need to be selected for different measurement systems. The measurement system proposed in this paper is classified as a metrological, repeatable, and automated measurement system, and is validated using analysis of variance (ANOVA). ANOVA is a standard statistical analysis technique, with the main evaluation indicators being GRR% and NDC. According to the AIAGMSA fourth edition standard, a measurement system is acceptable when GRR%≤10%. NDC≥5 indicates that the measurement system can distinguish differences between different measured objects.

GRR% quantitatively analyzes the contribution of different sources of variation in a measurement system by decomposing the variance of measurement data. The relationship between them can be expressed as:

(11) $$\sigma_{\mathrm{Total}}^{2}=\sigma_{\mathrm{Act}}^{2}+\sigma_{\mathrm{Reproducibility}}^{2}+\sigma_{\mathrm{Repeatability}}^{2}+\sigma_{\mathrm{IV}}^{2}$$

The first term in the formula represents the actual variation of the measured object; the second term represents the reproducibility variation; the third term represents the repeatability variation; and the last term represents the variation caused by the interaction. The total GRR variance, total variation, and percentage variation of the measurement system can then be calculated.

(12) $$\sigma_{\mathrm{GRR}}^{2}=\sigma_{\mathrm{Repeatability}}^{2}+\sigma_{\mathrm{Reproducibility}}^{2}+\sigma_{\mathrm{IV}}^{2}$$

(13) $$\sigma_{\mathrm{Total}}^{2}=\sigma_{\mathrm{Act}}^{2}+\sigma_{\mathrm{GRR}}^{2}$$

(14) $$GRR\%=\frac{\sigma_{\mathrm{GRR}}}{\sigma_{\mathrm{Total}}}\times 100\%$$

NDC represents the number of valid categories that a measurement system can distinguish; it is a statistical indicator of the system’s resolution.

Its calculation formula is:

(15) $$NDC=1.41\times \frac{\sigma_{\mathrm{Act}}}{\sigma_{\mathrm{GRR}}}$$

This paper uses Minitab 20 software to analyze three motion parameters of the vector nozzle: opening, deflection, and azimuth attitude. The results show that the GRR% and NDC of the measurement system are 2.81% and 7.69%, and 1.84%, 50, 18, and 76, respectively, indicating that the measurement system has high accuracy and stability. Finally, the variance analysis report for the three motion parameters was obtained, as shown in the figure below. Analysis of the X-plot and S-plot shows that all measurement points are within the control limits, indicating that the measurement system has stability and good repeatability. Furthermore, the results remain highly consistent among different operators, with no significant operator variation. The variation between measurement variables mainly originates from the variation between workpieces, and the error caused by the measuring instrument itself is very small. This demonstrates that the measurement system proposed in this paper has superior accuracy and reliability.

**Figure 14:**
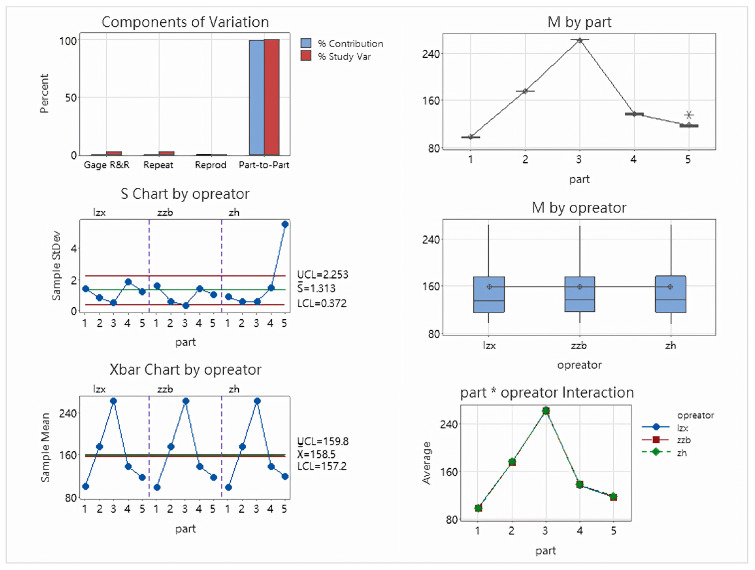
Opening ANOVA plot.

This paper uses Minitab software to analyze three motion parameters: nozzle opening, deflection, and azimuth attitude. This paper sets up three operators for each motion parameter and repeats the measurement of the five parameters ten times. The opening analysis table is shown in Table 1. The analysis of variance report is shown in Figure 14.

The opening analysis results show a Total Gage R&R of 2.81%, far below the 10% evaluation standard, indicating that the measurement system has high reliability for aperture measurement. Repeatability is 2.81% and Reproducibility is 0%, indicating minimal fluctuations in the measuring equipment and no significant differences between operators. The Part-to-Part ratio is 99.96%, and the NDC is 50, demonstrating that the measurement system can effectively distinguish the true differences between different samples. Various control charts (Xbar, S, Operator interaction chart, etc.) all exhibit good stability, no out-of-limit points, and no interaction effects, further validating the effectiveness and consistency of the measurement system for aperture measurement.

**Figure 15:**
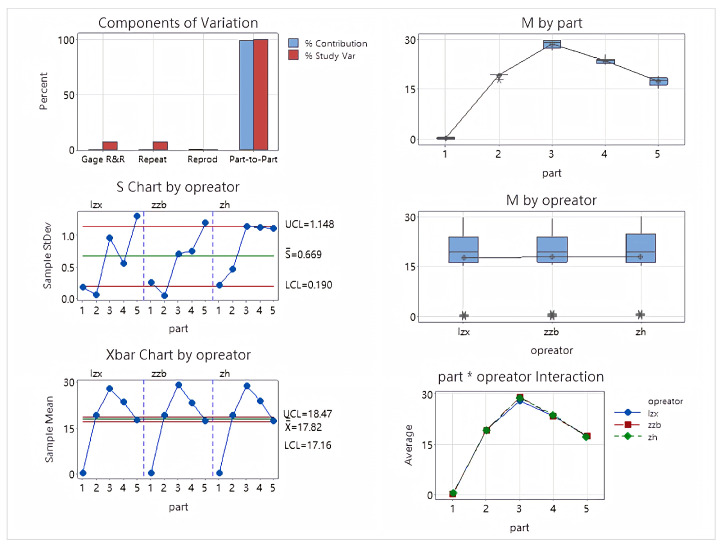
Deflection angle ANOVA plot.

Secondly, the deflection analysis table is shown in Table 2. The analysis of variance report is shown in Figure 15.

The deflection analysis results showed a Total Gage R&R of 7.69%, lower than the 10% evaluation standard, indicating that the measurement system has high reliability for deflection angle measurement. Repeatability was 7.69% and Reproducibility was 0%, indicating minimal fluctuations in the measurement equipment and no significant differences between operators. The Part-to-Part ratio was 99.70%, and the NDC was 18, demonstrating that the measurement system can effectively distinguish the true differences between different samples. Various control charts (Xbar, S, Operator interaction chart, etc.) all showed good stability, no out-of-limit points, and no interaction effects, further validating the effectiveness and consistency of the measurement system for deflection measurement.

**Figure 16:**
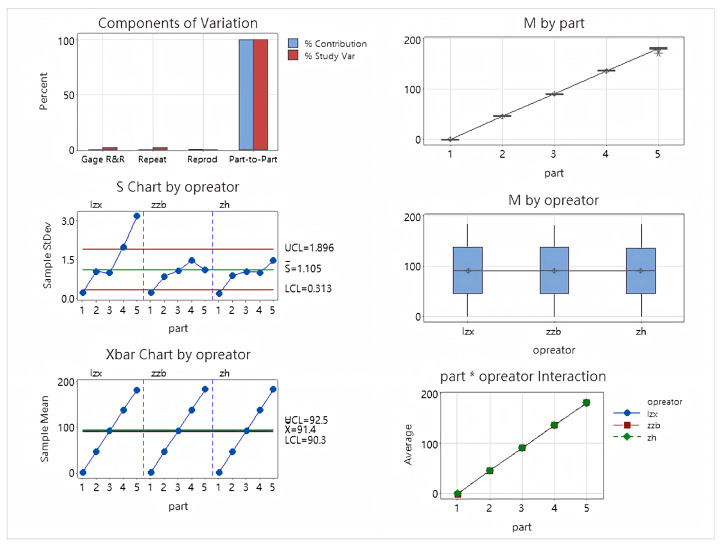
Azimuth ANOVA plot.

Finally, the azimuth analysis table is shown in Table 3. The analysis of variance report is shown in Figure 16.

The azimuth analysis results show a Total Gage R&R of 1.84%, lower than the 10% evaluation standard, indicating that the measurement system has high reliability for orientation angle measurement. Repeatability is 1.84% and Reproducibility is 0%, indicating minimal fluctuations in the measurement equipment and no significant differences between operators. The Part-to-Part ratio is 99.98%, and the NDC is 76, demonstrating that the measurement system can effectively distinguish the true differences between different samples. Various control charts (Xbar, S, Operator interaction chart, etc.) all exhibit good stability, no out-of-limit points, and no interaction effects, further validating the effectiveness and consistency of the measurement system for azimuth measurement.

## 5. Conclusions

In this paper, a multi-view stereo vision-based system for measuring the motion parameters of a vector nozzle is proposed, which features high accuracy, a large measurement range, and good stability. This paper designed a dual-bearing rotating nozzle and built a multi-view vision platform for experimental verification. A technical scheme of ‘ 3D reconstruction—point cloud segmentation—fitting measurement’ was proposed. Experimental results show that the system can achieve high-precision measurement. Furthermore, the stability and reliability of the proposed measurement system in measuring the motion parameters of the vector nozzle were demonstrated through MSA quantitative analysis. Although our method demonstrates good accuracy and stability, it still has certain limitations. Firstly, the proposed method has a high operating cost; currently, the system can only achieve offline real-time measurement and does not yet support online real-time measurement. Secondly, the system relies on a multi-view vision platform, resulting in high deployment complexity, and further optimization is needed for practical engineering applications. Future work could focus on algorithm lightweighting or researching more compact hardware configurations to achieve online real-time measurement capabilities.

## Figures and Tables

**Table 1:** Analysis of variance table of opening.

Source	StdDev (SD)	Study Var (6 × SD)	%Study Var (%SV)
Total Gage R&R	1.8135	10.881	2.81
Repeatablity	1.8135	10.881	2.81
Reproducibility	0	0	0
Operator	0	0	0
Part-To-Part	64.4532	386.719	99.96
Total Variation	64.4787	386.872	100

**Table 2:** Analysis of variance table of deflection.

Source	StdDev (SD)	Study Var (6 × SD)	%Study Var (%SV)
Total Gage R&R	0.8190	4.9140	7.69
Repeatablity	0.8190	4.9140	7.69
Reproducibility	0	0	0
Operator	0	0	0
Part-To-Part	10.6154	63.6926	99.7
Total Variation	10.6470	63.8818	100

**Table 3:** Analysis of variance table of azimuth.

Source	StdDev (SD)	Study Var (6 × SD)	%Study Var (%SV)
Total Gage R&R	1.3119	7.872	1.84
Repeatablity	1.3119	7.872	1.84
Reproducibility	0	0	0
Operator	0	0	0
Part-To-Part	71.4645	428.787	99.98
Total Variation	71.4766	428.859	100

## Data Availability

The data presented in this study are available on request from the corresponding author (The data are not publicly available due to institutional policy and ongoing research confidentiality).
